# Assessing the long-term economic impact of wheezing episodes after severe RSV disease in children from Argentina: a cost of illness analysis

**DOI:** 10.1136/bmjph-2024-000975

**Published:** 2024-07-30

**Authors:** Julia Dvorkin, Clint Pecenka, Emiliano M Sosa, Andrea Sancilio, Karina Dueñas, Andrea Rodriguez, Carlos Rojas-Roque, Patricia B Carruitero, Ranju Baral, Elisabeth Vodicka, Fernando P Polack, Romina Libster, Mauricio T Caballero

**Affiliations:** 1 Fundación Infant, Buenos Aires, Argentina; 2 Escuela de Bio y Nanotecnología, Universidad Nacional de San Martin Buenos Aires, Consejo Nacional de Investigaciones Cientificas y Tecnicas (CONICET), Buenos Aires, Buenos Aires, Argentina; 3 Center for Vaccine Innovation and Access, PATH, Seattle, Washington, USA; 4 Centre for Health Economics, University of York, York, UK; 5 Facultad de Ciencias Económicas, Universidad Nacional de la Plata, La Plata, Argentina

**Keywords:** economics, Epidemiology, Public Health

## Abstract

**Introduction:**

There is lack of available data on the economic burden of wheezing episodes after severe respiratory syncytial virus (RSV) infection. This study aimed to assess the cost incurred for wheezing episodes after a severe RSV infection in children from Argentina, considering both the public health system and societal perspectives.

**Methods:**

A prospective cohort was conducted from 2014 to 2022 to assess the cost of illness of wheezing episodes after severe RSV disease in children from Buenos Aires, Argentina. Direct medical and non-medical costs were estimated, along with indirect costs per episode and patient. Data pertaining to healthcare resource utilisation, indirect expenses and parental out-of-pocket costs were obtained. The overall cost per hospitalisation and health visits was calculated. Costs were quantified in US dollars using the average exchange rate on the specific date of data collection.

**Results:**

A total of 256 infants experienced severe RSV disease during their first year of life and were followed over a 5-year period in Buenos Aires. Overall, 150 children between 12 and 60 months presented 429 wheezing episodes. The median number of wheezing episodes per patient was 5 (IQR 3–7). The mean cost per wheezing episode was US$191.01 (95% CI 166.37 to 215.64). The total cost per episode of wheezing was significantly higher (p<0.001) in infants under 12 months of age (207.43, 95% CI 154.3 to 260.6) compared with older toddler. The average cumulative cost associated to wheezing per patient was US$415.99 (95% CI 313.35 to 518.63). Considering both acute RSV disease and long-term wheezing outcomes, the cumulative mean cost per patient was US$959.56 (95% CI 832.01 to 1087.10).

**Conclusions:**

This study reveals the economic impact of prolonged wheezing resulting from severe acute RSV infection on Argentina’s public health system and society. The estimates obtained serve as valuable inputs for informing cost-effectiveness analyses of upcoming RSV preventive interventions.

**WHAT IS ALREADY KNOWN ON THIS TOPIC**

- Multiple studies demonstrate the association between severe acute lower respiratory tract infections caused by respiratory syncytial virus (RSV) in infancy with long-term obstructive pulmonary disease such as recurrent wheezing or non-atopic asthma. Nevertheless, there is a lack of information regarding the economic impact of these frequent wheezing episodes in individuals who experienced hospitalisation due to RSV disease early in life, particularly in low- and middle-income countries. To address this gap, we conducted a prospective cohort study to ascertain the cost of illness associated with wheezing episodes in children during their initial 5 years of life following a severe RSV infection within a low-income population in Buenos Aires, Argentina.

**WHAT THIS STUDY ADDS**

- This study provides a comprehensive account of both medical and non-medical expenses associated with frequent wheezing episodes in childhood in low-income settings of Argentina, focusing on patients who experienced a severe RSV infection. Furthermore, we computed the total cost, encompassing the expenses associated with the initial severe RSV disease in those patients with long-term wheezing episodes.

**HOW THIS STUDY MIGHT AFFECT RESEARCH, PRACTICE OR POLICY**

- The data produced in this study are important for estimating the economic impact of forthcoming preventive measures against RSV in low- and middle-income countries through cost-effectiveness studies. Health decision-makers can leverage this information for future decisions on implementing preventive policies against RSV in infancy.

## Introduction

 Respiratory syncytial virus (RSV) is a ubiquitous pathogen that poses a considerable threat to infants and young children globally, causing millions of hospitalisations due to acute lung infection and more than 100 000 deaths every year.^1^,^4^ More than 90% of the fatal cases associated with this virus occur in contrasting settings of low- and middle-income countries (LMICs).^5^ In Argentina, the mortality rate for RSV is high, reaching more than 0.5 per 1000 live births, with more than 50% of the fatal cases taking place at home in the absence of medical attention.^6^,^9^

While the immediate clinical impact of severe RSV infections is well documented, there is an emerging recognition of the long-term sequelae that can follow an early and severe acute lower respiratory tract infection (ALRTI) caused by RSV, particularly the development of recurrent wheezing and asthma.^10^,^15^ Recurrent wheezing, characterised by episodic wheezing and breathing difficulties, can persist for years after an initial severe RSV infection and often requires ongoing medical care and treatment.^16 17^ These repeated episodes affect the quality of life for children and their families and impose a substantial economic burden on the healthcare system and society as a whole.^18 19^ In the USA, the combined financial expenses associated with various types of asthma amount to US$14 billion/year, comprising US$9.4 billion in direct expenditures and US$4.6 billion in indirect costs, which encompass missed school and workdays.^20^ Like prevalence rates, costs for asthma care vary significantly, with one study suggesting a cost ranging from $300 to $1300 per patient annually.^20^ On the other hand, costs related to recurrent wheezing in preschool children are estimated to range widely from US$1020 to US$29 000 per child per year in the USA and Canada.^21 22^ However, the economic impact of recurrent wheezing as an association of severe RSV disease has not yet been extensively explored in LMICs. While some research has been conducted on the clinical aspects of RSV infections and their associated costs in Argentina and other LMICs,^23 24^ there is a considerable lack of comprehensive assessments regarding the long-term effects of RSV infection on healthcare resource utilisation and associated costs.^25 26^

Given the possibility of upcoming preventive measures, it is imperative to estimate the cost of illness (COI) for long-term wheezing outcomes associated to a previous severe ALRTI due to RSV in LMICs.^4^ This is of utmost importance for several compelling reasons. To begin with, the majority of severe cases and fatalities occur within these nations, necessitating the swift deployment of preventive strategies to reduce the impact of RSV-related illnesses.^13 27^ Additionally, studies on the COI offer valuable insights into the financial strain caused by RSV, thereby revealing potential cost savings in the absence of this disease. Lastly, COI investigations contribute to a more holistic comprehension of the condition, encompassing its economic implications.^28^

The main objective of this study is to examine the COI associated with long-term wheezing episodes in children who have experienced severe RSV infections in Argentina during their first year of life. By shedding light on the economic dimensions of this complex issue, we aim to inform healthcare policy makers, promote effective preventive strategies and improve the overall well-being of affected children and their families.

## Methods

### Study design

We conducted a prospective cohort study for the evaluation of the COI associated with wheezing episodes that followed a severe RSV-ALRTI within the first year of life in children attended in two public hospitals of Buenos Aires, Argentina. The main study followed for 5 years patients under 12 months of age who were hospitalised due to RSV-ALRTI, allowing for a comprehensive assessment of the long-term economic impact of wheezing syndrome in this population. The analysis was carried out from both public health system and societal perspectives.^29^ This study adhered to the methodological considerations previously published for studies on the COI.^30 31^ Patients and the public were not involved in the design, conducting, reporting or dissemination plans of this study.

### Data source and study setting

This study employed data derived from a prospective cohort established at two public hospitals located in the southern region of Buenos Aires, Argentina. Both hospitals are comparable in terms of number of inpatient and paediatric intensive care unit (PICU) beds. The population accessing these hospitals is the same. The enrolment process for this cohort took place from May 2014 to August 2016, and the follow-up period was until June 2022. The cohort’s database encompasses details regarding the utilisation of healthcare resources, RSV diagnoses and demographic information of individuals who experienced ALRTI as previously described and detailed in the Instruments section and in online supplemental materials.^26^

### Local context

The healthcare system in Argentina is a mix of public, private and social security providers.^32^ It is decentralised, with each province managing its own healthcare services.^32^ The public healthcare system provides services to the entire population, while those with private insurance have access to a network of private clinics and hospitals.^32^,^34^ Challenges include disparities in healthcare quality between urban and rural areas and variations in access to specialised services.^8 35^ In the Buenos Aires Province, approximately 38% of the population does not have health coverage and relies only on the public healthcare system (https://redatam.indec.gob.ar/).^36^ Patients do not pay any out-of-pocket expenses for their care during their stay: food, diagnostic tests and treatments are all covered by universal health coverage.

### Instruments

For the collection of healthcare resources, data collection instruments were developed in REDCap as previously described.^26 37^ Parents or responsible adults were subject to multiple interviews conducted via telephone calls to gather information regarding their employment status, monthly income and itemised out-of-pocket expenses using research forms and uploading them to REDCap. For long-term wheezing outcomes, the study physicians filled out the research forms on each occasion when patients experienced respiratory exacerbation episodes and attended outpatient health clinics or inpatient paediatric wards. In instances where health visits did not occur, study members maintained periodic contact (every 2 months) with the families through phone calls, WhatsApp messages or email.

### Population

Infants aged 12 months or younger hospitalised with severe ALRTI were screened for eligibility. A comprehensive panel of 10 respiratory pathogens, including RSV, was identified in nasopharyngeal aspirates using real-time quantitative PCR (qPCR) in our research laboratory for each eligible patient.^6 9^ Those infants diagnosed of RSV by qPCR were included in this study.^38^ Severe ALRTI was defined as the presence of at least one manifestation of lower respiratory tract infection sign (cough, nasal flaring, indrawing of the lower chest wall, subcostal retractions, stridor, rales, rhonchi, wheezing, crackles or crepitations, or observed apnoea) plus hypoxaemia (peripheral oxygen saturation of <95% at room air) or tachypnoea (≥70 breaths per minute from 0 day to 59 days of age, and ≥60 breaths per minute at 60 days of age or older).^6 39 40^

Infants identified with severe RSV-ALRTI were tracked for a minimum of 5 years to monitor the presence of wheezing episodes (see online supplemental figure 1). The assessment of wheezing episodes was overseen by a study physician or periodically reported by parents through phone calls or face-to-face interviews using the International Study of Asthma and Allergies in Childhood questionnaire.^13 41^ Inclusion in the cost estimation required the presence of at least one documented episode of wheezing diagnosed by a physician following a severe RSV-ALRTI.

Loss of follow-up was deemed to occur if patients were unreachable through phone, email or WhatsApp, did not visit the primary care centre in the participant hospitals or relocated to another town outside the designated catchment area of the study (Metropolitan Area of Buenos Aires).

### Outcome of the study

The study’s objective was to determine the overall cost, considering both direct and indirect costs, associated with hospitalisations and healthcare visits due to wheezing episodes.^38^ All costs in this study were initially calculated in Argentine pesos (ARS) at the time data collection was obtained and subsequently converted to US dollars using the average exchange rate reported by the Central Bank of Argentina on that specific date.^42^

### Cost estimation

Medical cost estimation was based on the bottom-up costing method described elsewhere.^38^ In this approach, healthcare resources used were meticulously identified and quantified through the data collection instrument. The unit cost associated with each healthcare resource was extracted from the financial database and the account records of the two participating hospitals.^38^ It should be noted that since both hospitals are public, the costs are similar as they have standardised case costing procedures.

Through medical visits or phone calls, all 256 patients were followed up for at least 5 years and every wheezing episode was recorded along with the following information:

- Number of visits to the clinician per episode until recovery.
- Severity of the episode according to the Pulmonary Index Score (only for medical visits)^43 44^: in every visit, presence of wheezing, accessory muscle use, rales and oxygen saturation were documented and then the episode was classified under mild, moderate or severe ALRTI.
- Need for hospitalisation.

For outpatient episodes, if the severity of illness in the visit to the clinician was moderate, a standard treatment on the emergency room (ER) was established that included the administration of albuterol and supplementary oxygen with a Venturi mask, systemic corticosteroids and antipyretic drugs if necessary. The consultation fees for physicians and nurses were also considered in the total cost for outpatients.

For both outpatient and inpatient episodes, the total cost was disaggregated in the following components as previously described^38^: laboratory tests, labour cost, drugs, feeding, imaging diagnosis, supplies, oxygen supply, overhead and equipment depreciation.^38^ All these component costs were allocated as previously reported.^38^ The total cost was calculated per episode and per patient, considering that every subject included may have had one or more episodes of wheezing (with or without hospitalisation requirement).

To estimate the out-of-pocket expenditures, a two-part model was employed to address the substantial proportion of observations at zero combined with skewed positive outcomes (for non-zero).^45^ A similar approach has been used in a previous study.^46^ In the first part, three logit models were used to estimate the probability of incurring out-of-pocket expenditures for meals, commute and loss of income, respectively. Briefly, we modelled the natural log odds as a linear function of demographic (sex, age in months, patient’s weight, household income, parent’s education) and clinical (days of hospitalisation, number of wheezing episodes) covariates. Mathematically, we estimated the following equation:

$$logit\;\left(y\right)=ln\left(\frac{p}{1-p}\right)=\alpha +\beta X+Z\gamma \;+\varepsilon$$

where p is the probability of the outcome of interest y; X and Z are a subset of sociodemographic and clinical covariates; and ε is the error term with distribution ϵ∼N (0,1).^45^

In the second part, a generalised linear model with an identity link function and a gamma distribution for error terms was used to estimate the level of out-of-pocket expenditures for meals, commute and loss of income. This model was chosen instead of the conventional ordinary least squares (OLS) regression because cost data often exhibit significant skewness and adhere to a non-normal distribution, potentially leading to violations of the fundamental assumptions of OLS.^47 48^ The covariates included in the model were sociodemographic (sex, age in months, patient’s weight, household income, parent’s education) and clinical (days of hospitalisation, number of wheezing episodes). Lastly, the two parts were combined to estimate expected out-of-pocket expenditures by exploiting the basic rule of probability^46^:

E(y|X,Z)=Pr(y>0|X,Z)∗E(y|y>0, X,Z)

### Sample size and statistical analyses

Sample size was calculated using the following formula^49^:

$$\begin{array}{l} N={\left(\frac{precision^{2}}{Cofficient\;of\;Variation^{2}\times Z^{2}}+\frac{1}{Annual\;Cases}\right)}^{-1} \end{array}$$

Precision was established at ±10% of the mean cost, assuming a coefficient of variation of 0.5, a z-score of 1.96 and p (wheezing episodes after severe RSV) of 62%. The minimum sample size necessary for assessing the costs of RSV-associated long-term wheeze was determined to be 115 participants.

We analysed data using Stata (V.16.1, StataCorp, College Station, Texas). Descriptive statistics (frequency and per cent) were used to summarise demographics, clinical variables and healthcare resource utilisation. We applied for the Kolmogorov-Smirnov test to assess the normality of data. For normally distributed variables, we described data using means and SDs; while we described data using the median and IQR for non-normal distributed variables. We described the cost of recurrent wheezing episodes by using the mean together with its 95% CI. We used Student’s t-test to compare the average cost for outpatients and inpatients, and analysis of variance to compare the mean direct medical cost of a wheezing episode between different age groups based on reported wheezing frequency rates (0–12, 12–24 and >24 months of age).^11^

## Results

### Study population

Of the initially 470 patients aged 12 months or less screened for eligibility, 256 were confirmed to have severe RSV-ALRTI and enrolled in the study, and then followed for at least 5 years. The mean duration of follow-up for the 256 patients was 48.23 months (95% CI 44.50 to 51.99): 24 patients were lost to follow-up (6 during the 2nd and 3rd years and 18 during the 4th and 5th years). A total of 150 infants (58.6%) experienced at least one episode of wheezing after a severe ALRTI caused by RSV and were included in this analysis. The median number of wheezing episodes per patient reported during the follow-up was 5 (IQR 3–7, range 2–11). The majority of the episodes occurred within 2 years after the first severe RSV-ALRTI: the median time until the last episode of wheezing was recorded is 22 months (IQR 12–33.5).

We compared sociodemographic and clinical-biological variables between patients who developed repeated episodes of wheezing during the first 5 years of life and those who did not (table 1). Although both groups have similar characteristics, patients with no wheezing episodes more frequently lived in precarious housing (built mostly with tin, wood and/or mud) compared with controls (p=0.008). Across the population, the predominant employment status among parents was informal work, with a substantial monthly income variability. The study determined an average household income of US$632.8 per month, leaving more than 70% of the families situated below the poverty line according to Argentina’s income thresholds per family of four members.^50^

**Table 1:** Characteristic of the study population

	Non-wheezing* (n=106)	Wheezing (n=150)	P value
Sociodemographic			
Precarious housing, n/N (%)	10/106 (9.43)	3/150 (2.00)	**0.008**
No running water, n/N (%)	14/105 (13.33)	17/145 (11.72)	0.703
No sewage system, n/N (%)	59/104 (56.73)	95/146 (65.07)	0.182
Crowding (>3 inhabitants/room), n/N (%)	33/106 (31.13)	40/150 (26.76)	0.436
Incomplete parental education, n/N (%)†	58/93 (62.37)	78/124 (62.90)	0.935
Total family income, mean (SD)	699.68 (401.06)	619.18 (362.13)	0.163
Median household size, median (IQR)	5 (4–6)	5 (4–6)	0.442
Clinical and biological			
Female, n/N (%)	50/106 (47.17)	63/150 (42.00)	0.412
Age in months at first episode, mean (SD)	4.57 (2.71)	4.88 (3.21)	0.318
Diagnosis on first admission			
Bronchiolitis, n/N (%)	80/106 (75.47)	108/150 (72.00)	0.331
Pneumonia, n/N (%)	5/106 (4.72)	10/150 (6.67)	0.458
Pertussis-like syndrome, n/N (%)	4/106 (3.77)	4/150 (2.67)	0.366
Previous wheezing, n/N (%)	1/106 (0.94)	8/150 (5.33)	0.110
Sepsis	1/106 (0.94)	1/150 (0.65)	0.804
Healthcare-associated infection	1/106 (0.94)	–	–
Bronchopulmonary dysplasia	–	1/150 (0.65)	–
Other, n/N (%)	14/106 (13.21)	18/150 (11.84)	0.949
Admission to intensive care unit, n/N (%)	4/106 (3.77)	4/150 (2.67)	0.616
Length of stay in days, median (IQR)	6 (4–10)	7 (4–10)	0.579
Oxygen requirement in days, median (IQR)	5 (3–8)	6 (3–9)	0.551

Bold values represent a p value less than 0.05.

* No wheezing episodes after acute respiratory syncytial virus (RSV) illness.

† Incomplete high school or less for both parents.

IQRinterquartile rangeRSV-ARLTIrespiratory syncytial virus lower respiratory tract infectionSDstandard deviation

Overall, from the total of 429 episodes of respiratory exacerbations with wheezing, 324 received outpatient treatment, either through visits to the ER or paediatric primary care centres, and 105 needed hospitalisation in paediatric ward. A single wheezing episode may involve multiple health visits throughout its occurrence. No admission to PICUs or fatal events related to episodic wheezing were recorded in this study.

### Direct medical costs of wheezing episodes

Considering both outpatient visits and hospitalisations, the mean cost per episode (including all health visits) was calculated at US$185.00 (95% CI 160.33 to 209.67). No PICU admissions were registered for recurrent wheezing episodes. The mean direct medical cost for a severe wheezing episode necessitating hospitalisation, including all health visits before hospital admission, was US$528.32 (95% CI 454.01 to 602.64), as detailed in table 2. The aggregate cost for 105 hospital admissions due to wheezing episodes in 73 patients, requiring 716 hospital bed days (with a median stay of 5 days and an IQR of 3–8 days per episode), was US$60 729.81. In contrast, the average direct medical cost for a single episode not requiring hospital admission was US$85.92 (95% CI 80.51 to 91.34).

**Table 2:** Comparative analysis of itemised costs between inpatient and outpatient consultations for wheezing episodes

Costs	Inpatient(n=105)	Outpatient(n=324)
Laboratory test		
Mean	7.58	0.06
95% CI	5.64 to 9.53	0 to 0.15
Labour costs		
Mean	361.33	13.84
95% CI	303.88 to 418.78	13.04 to 14.64
Drug		
Mean	12.66	6.90
95% CI	10.43 to 14.89	6.53 to 7.28
Feeding*		
Mean	21.74	
95% CI	15.05 to 28.42	
Imaging diagnosis		
Mean	12.47	1.78
95% CI	11.18 to 13.76	1.31 to 2.24
Supplies		
Mean	4.04	0.62
95% CI	2.90 to 5.19	0.29 to 0.95
Oxygen supply		
Mean	43.05	0.07
95% CI	34.24 to 51.86	0.03 to 0.10
Overhead†		
Mean	8.87	10.70
95% CI	4.07 to 13.66	–
Equipment depreciation‡		
Mean	1.33	0.17
95% CI	1.22 to 1.43	0.12 to 0.21
**Total**	**Hospitalisation**	**One visit to the paediatrician**
Median	347.11	29.31
Mean	473.06	34.13
95% CI	399.16 to 546.97	32.60 to 35.66
**Total (for one episode)§**	**Allvisits+hospitalisation**	**All visits**
Median	425.40	84.12
Mean	528.32	85.92
95% CI	454.01 to 602.64	80.51 to 91.34

* Patient and one parent (either food or milk for patients under 6 months old), only for hospitalised patients.

† Bedding and linens, maintenance and administrative expenses.

‡ Includes equipment for mechanical ventilation, vital signs monitor, saturometer, infusion pump and diagnostic imaging equipment.

§ Patients may have more than one visit to the paediatrician during a wheezing episode or before hospitalisation.

When we analysed the cost by patient, the average cost for a hospitalisation was US$544.88 (95% CI 370.67 to 719.09), whereas the cost for an ambulatory health visit was US$58.67 (95% CI 48.13 to 69.22). Finally, we calculated the cumulative direct medical cost per patient, encompassing both severe RSV-associated ALRTI and the total long-term wheezing episodes, resulting in a mean of US$859.44 (95% CI 739.52 to 979.37).^38^

Similarly, as in the RSV-associated ALRTI COI estimation, labour costs of healthcare workers were a major cost driver of the overall cost (table 2), constituting between 40% and 76% of the total expenses.^38^

We have analysed each direct medical cost variable, considering age groups across all episodes (table 3), as well as variations in severity (online supplemental tables 1 and 2). We found that the total cost per episode of wheezing was significantly higher (p<0.001) in those infants younger than 12 months of age (207.43, 95% CI 154.3 to 260.6) compared with the subgroups of older toddlers. This difference can be partially explained by higher hospitalisation rates in this age group, leading to increased expenses in laboratory examinations, health worker costs and oxygen supply prices. No significant age-related differences were observed among children hospitalised due to severe obstructive wheezing exacerbations (online supplemental table 1). In contrast, when evaluating the costs associated with outpatient episodes, it became evident that the older subgroup of patients, specifically those aged over 24 months, exhibited a markedly higher total cost (mean $77.35, 95% CI 65.62 to 89.08, p<0.001), along with significantly elevated costs across each comparative variable (online supplemental table 2). Although fewer hospitalisations were recorded in children older than 2 years of age, the number of visits to the paediatrician, the severity of the wheezing episode and, therefore, the need of treatment in the ER before discharge were higher (p<0.001).

**Table 3:** Comparison of costs of wheezing episodes by age group

Costs	Wheezing episodes after severe RSV-ALRTI				
	Total episodesN=429	0–12 months(n=177)	12–24 months(n=152)	>24 months(n=100)	P value
Laboratory cost					
Mean	1.90	3.25	0.89	1.04	**0.0004**
95% CI	1.33 to 2.46	2.05 to 4.45	0.41 to 1.37	0.20 to 1.88	
Staff cost					
Mean	98.89	153.58	62.24	57.79	**0.000**
95% CI	79.02 to 118.76	111.43 to 195.72	42.86 to 81.62	33.85 to 81.74	
Drug cost					
Mean	8.31	8.31	7.51	9.54	0.072
95% CI	7.66 to 8.96	7.31 to 9.31	7.05 to 7.96	7.46 to 11.62	
Feeding cost*					
Mean	5.32	7.42	3.35	4.60	0.153
95% CI	3.48 to 7.16	4.31 to 10.53	0.74 to 5.96	0.47 to 8.72	
Imaging diagnostic cost					
Mean	4.39	5.01	3.55	4.58	0.142
95% CI	3.75 to 5.04	3.85 to 6.17	2.67 to 4.43	3.31 to 5.85	
Supplies cost					
Mean	1.46	1.69	0.90	1.89	0.117
95% CI	1.06 to 1.85	1.01 to 2.37	0.40 to 1.39	0.94 to 2.84	
Oxygen cost					
Mean	10.59	17.46	6.43	4.74	**0.0002**
95% CI	7.83 to 13.35	11.57 to 23.35	3.37 to 9.49	2.26 to 7.22	
Facilities cost					
Mean	10.25	10.18	9.59	11.38	0.523
95% CI	9.09 to 11.41	8.36 to 12.00	8.96 to 10.23	7.65 to 15.11	
Equipment cost					
Mean	0.45	0.53	0.37	0.44	0.091
95% CI	0.39 to 0.51	0.42 to 0.64	0.28 to 0.46	0.31 to 0.57	
Total cost					
Mean	141.56	207.43	94.84	96.00	**0.0001**
95% CI	116.22 to 166.90	154.29 to 260.57	69.47 to 120.20	62.43 to 129.57	

Bold values represent a p value less than 0.05

RSV-ALRTIrespiratory syncytial virus–acute lower respiratory tract infection

### Indirect costs and out-of-pocket expenses

Complete data on out-of-pocket expenses and indirect expenses were available for the families of 89 individuals (as shown in table 4). The average non-medical expenses associated with wheezing episodes in the cohort per individual were US$16.36 (95% CI 13.45 to 19.27), and per episode were US$4.89 (95% CI 4.27 to 5.50). The main driver of non-medical costs was food expenditures. Expenses were significantly higher in those hospitalised patients.

**Table 4:** Cost of non-medical expenses

Transport	n=109
Median	4.25
Mean	4.54
95% CI	4.12 to 4.97
Food	n=119
Median	5.52
Mean	7.85
95% CI	6.34 to 9.36
Indirect expenses	n=146
Median	2.53
Mean	4.14
95% CI	3.36 to 4.93
Total	n=89
Median	12.08
Mean	16.36
95% CI	13.45 to 19.27

### Total cost related to long-term wheezing episodes

Per wheezing episode, the total cost including medical and non-medical expenses was US$191.01 (95% CI 166.37 to 215.64). Based on the level of care, we observed that the total cost for hospitalisations was significantly higher (US$535.55, 95% CI 461.25 to 609.84) compared with outpatient episodes (US$92.34, 95% CI 86.74 to 97.94). In table 5, we presented the cumulative cost per individual, encompassing all wheezing episodes with and without the inclusion of acute RSV hospitalisations. Interestingly, the average total cost of all the wheezing episodes per child was US$415.99, with a maximum cost of US$5391.96, while adding RSV hospitalisation increased the cost to US$959.56 with a maximum of US$6024.35.

**Table 5:** Costs accumulated by individuals

Descriptive measures	Cumulative cost per individual	
	RSV-ALRTI+wheezing episodes	Wheezing episodes alone
Mean	959.56	415.99
95% CI	(832.01 to 1087.10)	(313.35 to 518.63)
Median	694.54	224.5
IQR	(511.72–1178.04)	(66.94–478.69)
Range	(189.83–6024.35)	(28.04–5391.96)

IQRinterquartile rangeRSV-ALRTIrespiratory syncytial virus–acute lower respiratory tract infection

## Discussion

This study explores the costs associated with long-term wheezing episodes after a severe RSV-ALRTI in the first year of life within a cohort treated at two public institutions in Buenos Aires, Argentina, based on primary data. This research estimates the comprehensive economic impact of both RSV disease and their lasting effects. The findings underscore the substantial economic burden imposed by RSV in Argentina, emphasising the significance of the study’s insights for future interventions against RSV globally. On analysing the cumulative number of outpatient and hospitalised wheezing episodes, it was observed that in over 50% of patients who experienced severe RSV infection in their first year, the total costs were double that estimated for a single hospitalisation due to ALRTI in public hospitals of Argentina.^38^ However, the ranges of cost estimates can vary significantly, especially in patients with severe respiratory infections and frequent exacerbations leading to numerous hospitalisations, where costs can be up to six times higher than the average.

Overall, there is scarce information regarding the economic consequences of wheezing episodes after severe RSV-bronchiolitis, particularly in LMICs.^13 27^ A recent study conducted in Colombia attempted to estimate the overall cost of RSV, accounting for long-term complications.^18^ The authors reported a daily cost per patient with long-term complications of US$840.52 (SD 189.79). However, the authors estimated this cost based on a decision model rather than direct estimations as we did in this approach.^18^ A retrospective study conducted in Japan highlighted that RSV produces a significant higher long-term economic burden when compared with other pathogens in both preterm and term children during their early years of life.^51^ The authors demonstrated a higher cumulative health cost associated with severe RSV disease. Nevertheless, the cumulative costs associated with severe RSV disease and its long-term effects in Japan are notably higher than those estimated in our cohort.^51^ Finally, the long-term utilisation of healthcare resources in the USA following infant RSV infection had a substantial impact. This influence persisted for at least 5 years after infection, with the majority occurring within the initial 2 years of life, aligning closely with the results obtained in this study.^28^

Argentina follows a vaccination policy that includes a comprehensive National Immunization Program, which provides free vaccines to the entire population, aiming to prevent a range of infectious diseases.^52^ In Argentina, RSV prevention policy is relevant. In this sense, the use of palivizumab was implemented several years ago and its use recommendation is guided by national health authorities for subgroup of infants at highest risk for severe RSV disease.^53^ Argentina has implemented diverse public health policies aimed at enhancing health outcomes and mitigating overall healthcare costs. These initiatives include vaccination campaigns, maternal and child health programmes, screenings for common diseases, promotion of primary healthcare services, advocacy for the use of generic medications and the implementation of the Remediar plan, ensuring free and equitable access to essential medications for individuals and communities with limited resources.^32 54^ Finally, for forthcoming public health decisions, it is vital that the Argentine government acquires accurate information on both the economic and epidemiological burdens of diseases, as outlined in this study. These data are indispensable for a comprehensive evaluation of health technologies. However, this study was conducted at two public hospitals situated in the southern Metropolitan Area of Buenos Aires. Hence, there is a need to broaden the investigation of costs, considering the perspectives of private and social security providers, which were not addressed in this study.

Our study has some limitations. Primarily, as the economic analysis was integrated into a more extensive prospective study, acquiring detailed and continuous information regarding families’ out-of-pocket and indirect expenses was not feasible. Furthermore, considering the prevalence of high unemployment and informal work in a population characterised by very low socioeconomic status, there is a potential underestimation of family expenses and lost workdays. To mitigate this challenge, we have applied a two-part model to address the substantial proportion of zero observation combined with biased positive results.^45 46^ However, even with these efforts, we believe our estimates of household expenses may still be underestimated. Regarding transportation, it is worth mentioning that most families live near the hospital within the capture area (median 3 km, IQR 2–5 km). These families primarily rely on bus transportation, which benefits from the state subsidies in Argentina. This may explain why food expenditure is the main driver of out-of-pocket expenses.

Lastly, it is essential to note that the Argentine government has also introduced various subsidies for medications, supplies and general expenses, potentially resulting in a reduction of the direct costs presented in this study.^54^ To gain a comprehensive understanding, it is imperative to analyse costs from private healthcare perspectives and consider variations across different regions of the country.

The recent endorsement of preventive interventions against RSV by the US Food and Drug Administration and the European Medicines Agency signifies a transformative shift in the paradigm of RSV disease.^55^ Various country government authorities now face the crucial task of assessing the feasibility of incorporating these preventive measures into routine medical practices, giving rise to diverse implementation scenarios.^55^ Hence, this study holds significant relevance, as the information it generates could allow us to understand that severe RSV disease has a short, medium and long-term impact from a clinical and economic perspective. The Argentine Ministry of Health has recently approved the implementation of the maternal vaccine against RSV, paving the way for a change in the burden of disease.^56^ Consequently, these insights will prove valuable in shaping future cost-effectiveness models.

The findings indicate that preventing RSV in the first year of life could significantly influence older children’s health and economic outcomes.

## supplementary material

10.1136/bmjph-2024-000975 online supplemental file 1

## Data Availability

Data are available in a public, open access repository.
